# A Novel Embolization Technique for Renal Hemorrhage Complication by Autologous Fat Tissue

**DOI:** 10.4274/balkanmedj.galenos.2019.2019.10.23

**Published:** 2020-02-28

**Authors:** Hasan Arı, Selma Arı, Veysi Can

**Affiliations:** 1 Clinic of Cardiology, Bursa Postgraduate Hospital, Bursa, Turkey

**Keywords:** Bleeding, coil/device/transcatheter, disease, embolization, renal artery intervention

## Abstract

The percutaneous nephrolithotomy method is the most used treatment option for urinary stone disease. Bleeding is the most feared complication of this method. Transcatheter coil or medical glue embolization is currently the used treatment option for this bleeding complication. The aim of this report to show the novel subcutaneous fat tissue embolization technique for percutaneous nephrolithotomy related bleeding complications. In these two cases, we treated the percutaneous nephrolithotomy related bleeding complication with subcutaneous fat tissue. Subcutaneous fat tissue was taken with subcutaneous fascia from the femoral site by 1 cm incision. This tissue to be used for embolization was passed on the 0.014 guidewire from the back end of this guidewire (like shish kebab). The fat tissue passed on the 0.014 wire was sent to the bleeding site through the guiding catheter and guideliner, over the 0.014 guidewire with the monorail balloon (as a pusher) placed behind this fat tissue. The subcutaneous fat tissue with subcutaneous fascia embolization was completely stopped the bleeding and fistula. We have successfully used a novel embolization technique for fat tissue embolization to the bleeding site (Ari technique). Subcutaneous fat tissue embolization with the novel embolization technique for percutaneous nephrolithotomy related bleeding is a safe and reliable treatment option.

## INTRODUCTION

Percutaneous nephrolithotomy (PCNL) treatment is a minimally invasive method in urinary stone disease. Bleeding is one of the most important complications in PCNL treatment. Bleeding incidence that was reported varies between 0.8% and 7.6% (1). Transcatheter coil or medical glue embolization is currently the most effective treatment option for PCNL related bleeding complications. Subcutaneous fat tissue embolization has used for coronary perforation treatment previously (2). However, subcutaneous fat tissue embolization has not been used until now for renal bleeding complications.

In these two cases, we performed subcutaneous fat tissue embolization with a novel technique for PCNL related bleeding complications.

## TECHNIQUE

## CASE 1

Before presentetion consent form was filled out by all participants. A 53 year-old male patient who underwent extracorporeal shockwave lithotripsy for 3 sessions and underwent PCNL from the right kidney 7 years ago, was presented to the urology department with right lumbar pain. As a result of the diagnostic tests, a few stones (largest one is 2 cm) were found at the lower pole of the right kidney. This patient was hospitalized in the urology clinic. The kidney stones was removed from the lower pole with two entry by PCNL and the procedure was terminated with inserting 14 F reentry nephrostomy catheter. Three days after the procedure the reentry nephrostomy catheter was removed.

**Figure 1:**
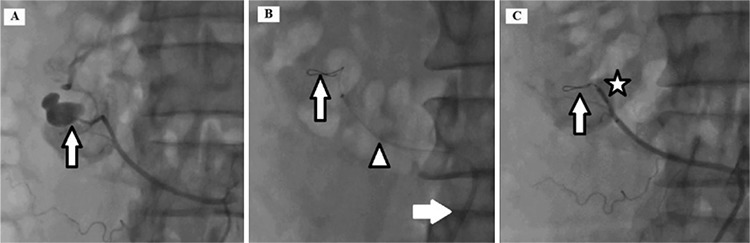
(a) Accessory renal artery hemorrhage and arteriovenous fistula (arrow), (b) 0.014 guidewire at the bleeding segmental artery (arrow), 5.5 F GuideLiner catheter (arrowhead), 6 F right guiding catheter (white arrow), (c) Stopped bleeding (arrow), inflate 2.5x15 mm balloon (star).

**Figure 2:**
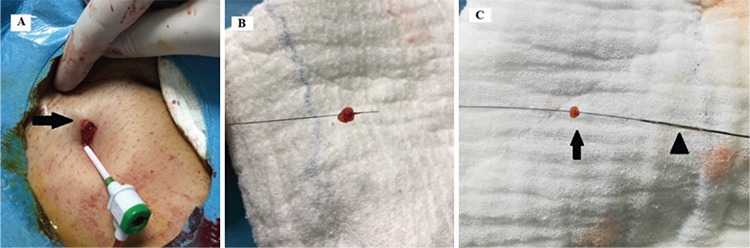
(a) Femoral site about 1 cm incision (arrow), (b) fat tissue on the guidewire (like shish kebab), (c) fat tissue (arrow) and monorail balloon (as a pusher) (arrowhead) on the guidewire.

**Figure 3:**
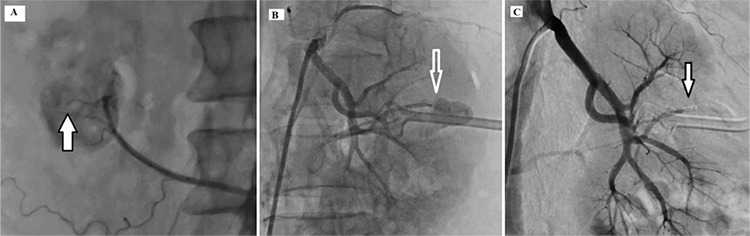
(a) Stopped bleeding with fat tissue embolization (arrow), (b) renal segmental artery hemorrhage (arrow), (c) stopped bleeding with fat tissue embolization (arrow).

The hemoglobin value was 14.5 g/dL before the procedure. Seven days after the procedure hemoglobin was decreased to 7.5 g/dL due to hematuria. The patient was treated with 2 unit erythrocyte suspension (ES) and 1 unit fresh frozen plasma (FFP) but hematuria was continued. Ten days after the PCNL, accessory renal artery angiography revealed hemorrhage and arteriovenous fistula in the lower pole segmental artery of the right kidney (Figure 1A, Video 1). A 6 F right guiding catheter (Mach 1, Boston Scientific, USA) was used to cannulate the right accessory renal artery. The 0.014 ChoICE Floppy guidewire (Boston Scientific/Scimed Maple Grove MN) was sent to the bleeding segmental artery, for the embolization of the fat tissue to the right place, 5.5 F GuideLiner catheter (Vascular Solutions Inc., Minneapolis, MN, USA) was advanced through the 6 F right guiding catheter and over the 0.014 guidewire to the segmental artery that was bleeding (Figure 1B, Video 2). The 2.5x15 mm monorail balloon (Invader PTCA balloon, Alvimedica, Turkey) sent over the 0.014 wire was inflated and bleeding was stopped and fistula was closed (Figure 1C, Video 3). After verification of the bleeding and fistula artery with balloon occlusion, fat tissue embolization was decided. For subcutaneous fat tissue embolization, about 3x3 mm subcutaneous fat tissue was taken with subcutaneous fascia from the femoral site by 1 cm incision (Figure 2A). Subcutaneous fat tissue with subcutaneous fascia to be used for embolization was passed on the 0.014 guidewire, from the back end of this guidewire (like shish kebab) (Figure 2B). The fat tissue passed on the 0.014 wire was sent to the bleeding site through the guiding catheter and guideliner, over the 0.014 guidewire with the monorail balloon (as a pusher) placed behind this fat tissue (Figure 2C, Video 4). Embolization completely stopped the bleeding and fistula (Figure 3A, Video 5). Because heparin was not used during these procedures the catheter system was flashed with saline at short time intervals to prevent thrombus formation. This embolization technique was named the “Ari technique”.

After this procedure the patient was transferred to the urology service. One unit ES is given to the patient after the procedure and hemoglobin value was increased to 9.6 g/dL. The patient's hemodynamic and biochemical parameters were stable and he was discharged 2 days after the procedure. The patient was asymptomatic at follow-up for six months. The patient's hemoglobin value was 12.5 g/dL and the creatinine value was 0.98 mg/dL. At the 6^th^ month ultrasonography, the right kidney size was slightly smaller than the left and the lower pole cortex was thinner than the mid and upper pole cortex.

## CASE 2

Before presentetion consent form was filled out by all participants.A 67-year-old female patient presented to the urology department with left lumbar pain. As a result of the diagnostic tests, 1.5 cm stone was found in the left kidney. The kidney stone was removed as unblocked from the middle column by PCNL and the procedure was terminated with inserting 16 F reentry nephrostomy catheter. The hemoglobin value was 10.1 g/dL before the procedure. After the procedure hemoglobin was decreased to 6.9 g/dL due to hematuria and bleeding from the nephrostomy catheter.

The patient was treated with 3 unit ES and 1 unit FFP but bleeding was continued. Two days after the PNL, renal angiography revealed hemorrhage in the middle pole segmental artery of the left kidney (Figure 3B, Video 6). A 7 F right guiding catheter (Mach 1, Boston Scientific, USA) was used to cannulate the left kidney. The 0.014 ChoICE Floppy guidewire (Boston Scientific/Scimed Maple Grove MN) wire was sent to the bleeding segmental artery, and the 2.5x15 mm monorail balloon (Invader PTCA balloon, Alvimedica, Turkey) sent over the 0.014 wire was inflated and bleeding was stopped (like case 1). After confirming the bleeding artery, it was decided to perform subcutaneous fat tissue embolization. For subcutaneous fat tissue embolization, about 3x3 mm subcutaneous fat tissue with subcutaneous fascia was taken from the femoral site by 1 cm incision (like case 1). For the embolization of this size of fat tissue to the right place, 6 F GuideLiner catheter (Vascular Solutions Inc., Minneapolis, MN, USA) was advanced through the 7 F right guiding catheter and over the 0.014 guidewire to the segmental artery that was bleeding with balloon anchor. Subcutaneous fat tissue with subcutaneous fascia to be used for embolization was passed on the 0.014 guidewire, from the back end of this guidewire (like shish kebab). The fat tissue passed on the 0.014 wire was sent to the bleeding site through the guiding catheter and GuideLiner, over the 0.014 guidewire with the monorail balloon (as a pusher) placed behind this fat tissue. Embolization completely stopped the bleeding (Figure 3C, Video 7). We confirmed the embolization technique “Ari technique” success and safety with the second case.

After this procedure the patient was transferred to the urology service. One unit ES is given to the patient after the procedure and hemoglobin value was increased to 8.9 g/dL. The patient's hemodynamic and biochemical parameters were stable and she was discharged 3 days after the procedure. The patient was asymptomatic at follow-up for six months. The patient's hemoglobin value was 10.4 g/dL and the creatinine value was 0.84 mg/dL. At the 6th month ultrasonography, the left kidney mid pole cortex was thinner than other kidney cortex.

## DISCUSSION

In these two cases, we presented the percutaneous treatment of PCNL related bleeding complication by subcutaneous fat tissue and a new technique. This is the first case in literatüre that percutaneous treatment of PCNL related bleeding complication by subcutaneous fat tissue with subcutaneous fascia. In addition, the technique used in the subcutaneous fat tissue embolization was introduced into the literature as a new embolization technique.

PCNL is the preferred interventional procedure for the safe and effective treatment of kidney stones (3). Bleeding, pseudoaneurysm and arteriovenous fistula are the most serious complications of PCNL. Blood transfusion may be required in 11%-17% of patients due to bleeding complications after PCNL (4). Percutaneous embolization is required in patients whose bleeding despite blood transfusion after PCNL. Fortunately, less than 1% of patients with PCNL require percutaneous embolization (5,6).

Bleeding after PCNL is presented within the first 24 hours or 20 days. The mean time to the onset various between 6 and 8 days (5,7). If bleeding after PCNL cannot be controlled with conservative treatment, angiography is used both as a diagnostic and therapeutic modality. Renal embolization is currently considered the most appropriate technique in the treatment of vascular complication of percutaneous renal procedures (8,9,10). Endovascular technique have a lower complications rate than surgical management. Various embolic agent are available to treat vascular complications of PCNL including coils, particulate agent and liquid agents. They can be used either alone or as complementary tools (11).

The use of subcutaneous fat tissue in the treatment of bleeding complications during percutaneous interventions is an autologous, easily accessible and inexpensive method. This method has been used many times in cases of type 5 coronary perforation (2,12). The efficacy and safety of this treatment modality have been shown in cases of type 5 coronary perforation (2,12). It has been shown in our cases that the bleeding complication caused by PCNL can be treated effectively and safely with subcutaneous fat tissue with subcutaneous fascia.

The methods used in the standard treatment of bleeding complications caused by PCNL are coil or glue embolization. These treatment modalities are expensive methods requiring different microcatheter and materials. In addition, these materials may not be available in all laboratory in all times. Guiding catheter, 0.014 wire, monorail balloon, extension catheter that we used in our technique are present in every interventional angiography laboratory. Subcutaneous fat tissue is already taken from the patient complicated with bleeding. In previous applications, microcatheter was used for coronary embolization of subcutaneous fat tissue. However, large-scale microcatheters are needed for the embolization of fat tissue to the correct location. This makes difficult the embolization process and correct point embolization. In our technique, there is no need for microcatheters, the point where 0.014 guidewire is position is embolized by monorail balloon over this wire.

In the standart tecnique there is no guidewire in the embolization with microcatheter, so that during the procedure microcatheter may move backwards, that may cause embolization of the material to undesired areas. In case of emergency, such as distal coronary perforation, fat tissue embolization through the microcatheter may require a long guidewire or elongation wire, which leads to the loss of the guidewire in the bleeding area and the loss of time, resulting in tamponade development. In our technique, the embolization can be achieved quickly without the guidewire ever being moved. In addition, in our technique, materials such as small graft fragments can be rapidly embolized to the correct point (bleeding area). The risk of infection and allergic reaction in the autholog subcutaneous tissue will also be low. This technique is an inexpensive technique that does not require an extra cost in terms of the materials used.

In this technique, operator should be careful not to disintegrate the fat tissue on the wire. For prevention to such a problem, we took the fat tissue with the subcutaneous tissue. The other limitations of this tecnique are, the material is radiolucent and could not repositioned after released over the 0.014 wire. Some contrast can inject to the subcutaneous tissue for radiopacity of the tissue. At the same time this embolization technique will allow a treatment option of complications such as bleeding and fistula with different materials. Small grafts and silicon pieces (like fat tissue) can be used to treat fistula and bleeding complications with this technique.

Subcutaneous fat tissue embolization is a safe and reliable option for PCNL related bleeding complications. This new embolization technique can be used safely and effectively in the treatment of complications such as bleeding and fistula.

**Video 1.** Accessory renal artery hemorrhage and arteriovenous fistula.

**DOI: 10.4274/balkanmedj.galenos.2019.2019.10.23.video1**

**Video 2.** Advancing GuideLiner catheter to bleeding accessory renal artery.

**DOI: 10.4274/balkanmedj.galenos.2019.2019.10.23.video2**

**Video 3.** The bleeding was stopped with 2.5x15 mm monorail balloon inflation.

**DOI: 10.4274/balkanmedj.galenos.2019.2019.10.23.video3**

**Video 4.** Fat tissue was pushed with monorail balloon to bleeding site and 0.014 guidewire take back and fat tissue was released.

**DOI: 10.4274/balkanmedj.galenos.2019.2019.10.23.video4**

**Video 5.** Stopped bleeding with fat tissue embolization.

**DOI: 10.4274/balkanmedj.galenos.2019.2019.10.23.video5**

**Video 6.** Left kidney middle pole renal artery bleeding.

**DOI: 10.4274/balkanmedj.galenos.2019.2019.10.23.video6**

**Video 7.** Stopped renal artery bleeding with fat tissue embolization.

**DOI: 10.4274/balkanmedj.galenos.2019.2019.10.23.video7**
